# Facing the River Gauntlet: Understanding the Effects of Fisheries Capture and Water Temperature on the Physiology of Coho Salmon

**DOI:** 10.1371/journal.pone.0124023

**Published:** 2015-04-22

**Authors:** Graham D. Raby, Timothy D. Clark, Anthony P. Farrell, David A. Patterson, Nolan N. Bett, Samantha M. Wilson, William G. Willmore, Cory D. Suski, Scott G. Hinch, Steven J. Cooke

**Affiliations:** 1 Fish Ecology and Conservation Physiology Laboratory, Department of Biology and Institute of Environmental Sciences, Carleton University, Ottawa, Ontario, Canada; 2 Australian Institute of Marine Science, Townsville, Queensland, Australia; 3 Department of Zoology, University of British Columbia, Vancouver, British Columbia, Canada; 4 Fisheries and Oceans Canada, Science Branch, Pacific Region, Cooperative Resource Management Institute, School of Resource and Environmental Management, Simon Fraser University, Burnaby, British Columbia, Canada; 5 Pacific Salmon Ecology and Conservation Lab, Department of Forest and Conservation Sciences, University of British Columbia, Vancouver, British Columbia, Canada; 6 Institute of Biochemistry, Department of Biology, Carleton University, Ottawa, Ontario, Canada; 7 Department of Natural Resources and Environmental Science, University of Illinois, Urbana, Illinois, United States of America; Pacific Northwest National Laboratory, UNITED STATES

## Abstract

An improved understanding of bycatch mortality can be achieved by complementing field studies with laboratory experiments that use physiological assessments. This study examined the effects of water temperature and the duration of net entanglement on physiological disturbance and recovery in coho salmon (*Oncorhynchus kisutch*) after release from a simulated beach seine capture. Heart rate was monitored using implanted electrocardiogram biologgers that allowed fish to swim freely before and after release. A subset of fish was recovered in respirometers to monitor metabolic recovery, and separate groups of fish were sacrificed at different times to assess blood and white muscle biochemistry. One hour after release, fish had elevated lactate in muscle and blood plasma, depleted tissue energy stores, and altered osmoregulatory status, particularly in warmer (15 vs. 10°C) and longer (15 vs. 2 min) capture treatments. A significant effect of entanglement duration on blood and muscle metabolites remained after 4 h. Oxygen consumption rate recovered to baseline within 7–10 h. However, recovery of heart rate to routine levels was longer and more variable, with most fish taking over 10 h, and 33% of fish failing to recover within 24 h. There were no significant treatment effects on either oxygen consumption or heart rate recovery. Our results indicate that fishers should minimize handling time for bycatch and maximize oxygen supply during crowding, especially when temperatures are elevated. Physiological data, such as those presented here, can be used to understand mechanisms that underlie bycatch impairment and mortality, and thus inform best practices that ensure the welfare and conservation of affected species.

## Introduction

Recent studies suggest climate warming is affecting the distribution and phenology of fishes [1,2], yet the relatively inflexible life history of some species means that changes in distribution may be limited and thermal tolerance must keep pace with the warming environment [3]. In Pacific salmon (*Oncorhynchus* spp.), spawning stream fidelity and fixed reproductive schedules mean that fish have little or no choice about what water temperatures or fisheries they will encounter during upstream spawning migrations. High river temperatures (e.g., 18+ °C; [4]) can presumably act as a selective force because they cause mortality via disrupted physiological homeostasis or acceleration of pathogen development [4,5,6,7,8]. A variable portion of Pacific salmon intercepted by commercial, aboriginal, and recreational fisheries will be released or escape (e.g., [9,10]). The strategy of releasing certain species or populations for conservation purposes hinges on post-release recovery and high survival rates for fish exposure to what is an acute exercise stressor (capture and handling). The interaction between water temperature and fisheries capture stressors is of increasing relevance to management, as salmon-bearing rivers are projected to continue to warm [3,11,12].

A paradox of fisheries science is that while warmer water correlates positively with post- release mortality [13,14], it can also accelerate physiological recovery [15,16,17,18]. Fisheries capture typically elicits exhaustive exercise, hypoxia, injury, and a neuroendocrine stress response [19,20,21,22], which combine to cause rapid physiological changes from which the animal must recover. Physiological recovery profiles of exhaustively exercised and recreationally-angled fish have been well documented in the literature [23,24], but few studies have done the same for commercial fishery scenarios (i.e., bycatch, but see [25]).

Physiological recovery profiles have traditionally focused on plasma and muscle tissue analyses (e.g., [15,25,26], but some attention has been given to cardiorespiratory function [27,28,29]. A recent study found that heart rate (*f*_H_) required an extended period (~16 h) to return to baseline following fisheries-related capture stressors in free-swimming coho salmon (*O*. *kisutch*) implanted with heart rate data loggers [28]. Though a small number of papers have described heart rate responses to recreational angling (i.e., [27,30]), few have used cardiac measures to monitor recovery from fisheries capture because such measures normally require that the fish are tethered to recording equipment (e.g., to measure ECG or blood flow in the ventral aorta). Thus, sublethal fitness effects of fisheries capture are rarely considered [31]. Indeed, for in adult migrating salmon, extended cardiorespiratory recovery could be a particular concern, potentially diverting a significant amount of their finite energy stores away from migration, gonad development, and spawning.

Of the seven Pacific salmon species found in British Columbia, Canada, one of the least abundant are coho salmon, which includes an endangered interior Fraser River population (hereafter termed interior Fraser coho) that are required by regulation to be released alive when caught incidentally [32]. Field-based studies have examined factors influencing coho salmon post-release survival in the marine environment (gillnets—[33]; troll fisheries—[34]) and in freshwater [32]. Recent research has been aimed at understanding delayed mortality of interior Fraser coho released from the aboriginal beach seine fishery, which fish encounter during their upstream migration [32,35]. In those field studies it was not possible to assess physiological recovery, how handling time (i.e., time entangled in the seine net) affected physiological impacts or recovery, or the extent to which water temperature can modulate such effects. It is not possible to study interior Fraser coho in an experimental setting due to conservation concerns, but other populations can act as surrogates.

Here, we use Chilliwack River coho salmon to examine physiological recovery profiles after release from a beach seine capture simulation. We conducted the simulation using two temperatures (10 and 15°C) and two stressor durations (2 or 15 min seine net entanglement). We focused on monitoring the relative effects of different capture treatments on heart rate, oxygen consumption rate, and a suite of white muscle and blood plasma indices of metabolic and osmoregulatory status. The involvement of the authors in recent research on bycatch in the beach seine fishery [32] enabled the use of a realistic fishing simulation and an experimental design that can help answer questions that could not be addressed in the field—specifically, the interactive effects of temperature and entanglement time on physiological disturbance and recovery. By unveiling mechanisms that underlie impairment and mortality, physiological data from controlled experiments can inform bycatch management and handling practices and assist with interpretation of trends observed in field studies.

## Methods

### Study site and animals

**Fig 1:**
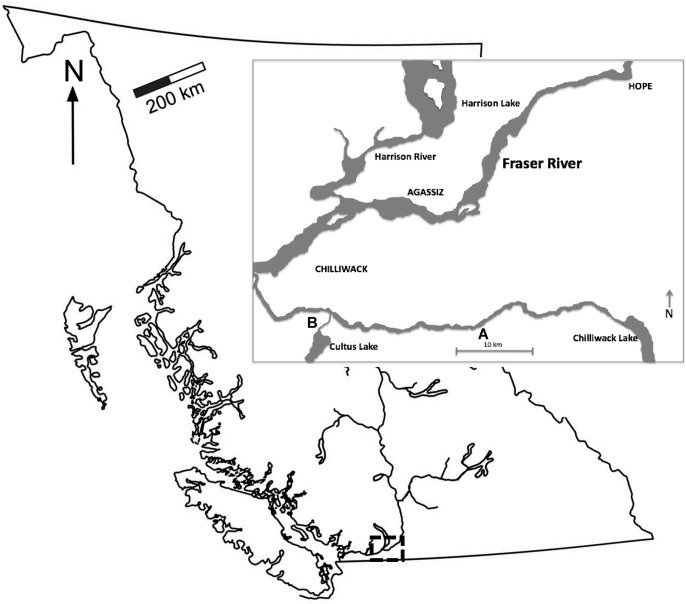
Map showing where the study was conducted in the Chilliwack River watershed, which is a part of Fraser River watershed of British Columbia, Canada. Fish were captured at the Chilliwack River Hatchery (A) and transported to Cultus Lake Laboratory (B) for experiments.

The fish used in this study were adult coho salmon from the Chilliwack River Hatchery (see Fig 1). These fish (mean ± standard deviation fork length = 62.5 ± 4.4 cm, mass = 2.92 ± 0.61 kg) had completed their 125 km upstream migration from the ocean to the hatchery where they had been reared and released ~1.5 years prior. Between 14/10 and 26/10/2011, fish were dip-netted from a concrete raceway at the hatchery and transported 22 km in aerated 8–10°C river water to Cultus Lake Laboratory (CLL; Fig 1) for experimentation. At CLL, fish were held in the transport tank, within which dissolved oxygen was maintained between 85–120% saturation, and were dip-netted individually for surgery prior to transfer into either of two large, circular concrete ponds (5.3 m diameter). The concrete ponds, in which fish were held post-surgery (see below), were sectioned off by wooden frames lined with 5 cm diameter stretch beach seine mesh so that fish were kept in one half of the pond. Fresh cold water was continuously pumped into both ponds via an intake at 15 m depth in nearby Cultus Lake. Water in each pond was 60 cm deep, and each pond was serviced by three large air stones that maintained air saturation >90%. An additional submersible pump was used to create a circular flow within each pond (~ 10 cm s^-1^).

### Experimental protocol

**Fig 2:**
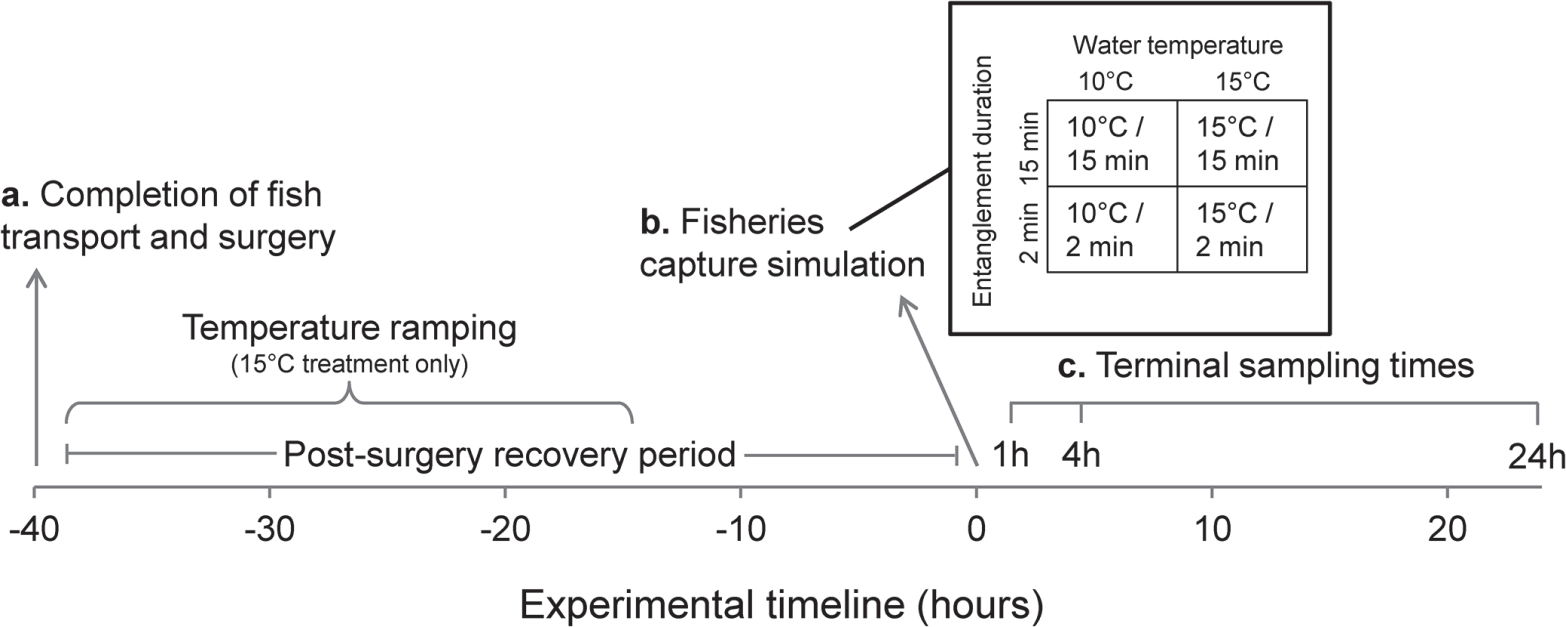
The experimental timeline that was repeated three times for each temperature. Letters correspond to sections in *Methods*.

The experiment, outlined in Fig 2, was replicated three times for both the warm and cold test temperatures. Terminal sampling occurred at three time points (1, 4, and 24 h) after the fisheries simulation and involved three separate groups of fish that were separated into net pens within the pond at the end of the capture simulation. The 24 h group was surgically implanted with data loggers and tagged with spaghetti tags while the other two groups were only spaghetti-tagged.

#### a. Fish surgery, transfer to experimental ponds, and temperature increase

Fish were dip netted from the transport tank and anesthetized with a knockout dose of 100 mg L^-1^ tricaine methanesulfonate (MS-222, Sigma Aldrich, St. Louis, MO) buffered with 200 mg L^-1^ NaHCO_3_ in Cultus Lake water (8.5–10°C). Fish remained in the anesthetic bath until they lost equilibrium and their opercular movement slowed (~5 min), at which point they were weighed and brought to the surgery bench where their gills were continuously irrigated with a well-aerated maintenance dose of anesthetic (70 mg L^-1^ MS-222 with 140 mg L^-1^ NaHCO_3_). The fish were kept prone for insertion of a uniquely numbered spaghetti tag (Floy Tag & Mfg. Inc., Seattle, WA, USA) through the dorsal musculature, just anterior to the dorsal fin, tied with a simple double reef knot. Next, a custom-made data logger (23 g in air, coated in biocompatible silicon, University of Tasmania, Australia) was surgically implanted into the intraperitoneal cavity as previously described [36]. Briefly, the logger was inserted through a 3–4 cm incision and uterine forceps were used to guide the anterior-most electrode sensor as close as possible to the pericardial cavity, ventral to the liver. The logger (programmed to turn on and record ECG and temperature for 10 s every 6 min) was loosely sutured with one suture to the peritoneal wall to prevent it from moving post-surgery. The incision was closed using five or six sutures tied into square knots (size 0 monofilament PDS II absorbable sutures, 36 mm ½ circle reverse cutting needle; Ethicon, Somerville, NJ). After incision closure and post-surgery revival in a freshwater-filled container, fish were released into an experimental pond.

The remaining fish that had been transported from Chilliwack Hatchery were anesthetized (as above) before being briefly transferred to a water-filled (without maintenance anesthetic) and padded V-shaped sampling trough for spaghetti tag insertion (as above). Fish were then allowed to complete their revival from anesthesia in the experimental pond. Including data logger-implanted fish, ~35 fish were tagged and placed into each of the two ponds (~10–12 each for 1 h, 4 h, 24 h sampling groups) and allowed to recover over two nights (40–45 h, see Fig 2) before the capture simulation.

Once all tagged fish were in the experimental ponds, the temperature in one of the two ponds was increased by supplementing the deep Cultus Lake water input with water that was run through a boiler system. Water inputs were adjusted to increase the warm treatment pond to ~15°C within 24 h after transfer, while the other pond was supplied only with deep Cultus Lake water (~10°C). Both temperatures are ecologically relevant in the context of coho salmon upriver migrations and the occurrence of harvest fisheries.

#### b. Capture simulation

A section of beach seine netting was used to gradually corral all fish to one corner of the pond. Once fish were corralled, the net was drawn under and around the fish such that they were pursed and could be pulled up onto a wooden platform that was dropped into the pond after corralling began. The platform was ~ 55 cm from the bottom of the pond and 1 × 1.5 m across, resulting in high crowding and a water depth of ~5 cm on the platform—conditions comparable to those in real beach seine fisheries when the seine is pulled into the beach (see [32] for photos). Once fish were crowded on the platform for 2 min, approximately half of the fish were removed (identifiable by unique spaghetti tags) and rapidly transferred using knotless nylon dip nets to one of three net pens within the pond. The three net pens were used to hold fish to be euthanized in separate groups at three time points (Fig 2). After 15 min of entanglement, the remaining fish were transferred into their respective net pens. Dissolved oxygen in the water on the crowded platform within the fishing net declined from ~90% air saturation to 50–60% saturation by the final minutes of the 15 min simulation. In real beach seine fisheries oxygen levels can decrease by 20–60% during sorting [32]. Thus, the capture stressor involved a stress response and exercise during corralling and netting, followed by confinement in shallow water with declining oxygen content.

#### c. Terminal sampling

Physiological sampling was carried out for each of the three pens at 1, 4, and 24 h after the initiation of the capture stressor (i.e., when the experimenters entered the pond to begin corralling fish; Fig 2). All fish were rapidly dip netted from their net pen and sacrificed by cerebral percussion within 30 s of the start of dip netting. Blood samples (1–2 mL) were then drawn within 3 min from the caudal vasculature of each fish using 21-gauge needles and heparinized vacutainers (3 mL with lithium heparin; BD, Franklin Lakes, New Jersey). Simultaneously, sections of white muscle were cut from the left side of each fish (within 5 min of being sacrificed), 1–2 cm above the lateral line anterior to the dorsal fin. Muscle samples were pressed firmly between a set of metal clamps that had been cooling in liquid nitrogen, and then stored in liquid nitrogen until later transfer to a -80°C freezer. Tubes containing blood samples were put immediately into a water-ice slurry and within 20 min they were centrifuged at 7,000 *g* for 5 min. The separated blood plasma was stored in liquid nitrogen before eventual transfer to a -80°C freezer. After physiological sampling was complete, fish were measured (fork length, FL, nearest cm) and sex was verified by examining gonads. In the case of 24 h fish, data loggers were removed, scrubbed clean in freshwater, and immersed in povidone-iodine for sterilization prior to re-use.

**Fig 3:**
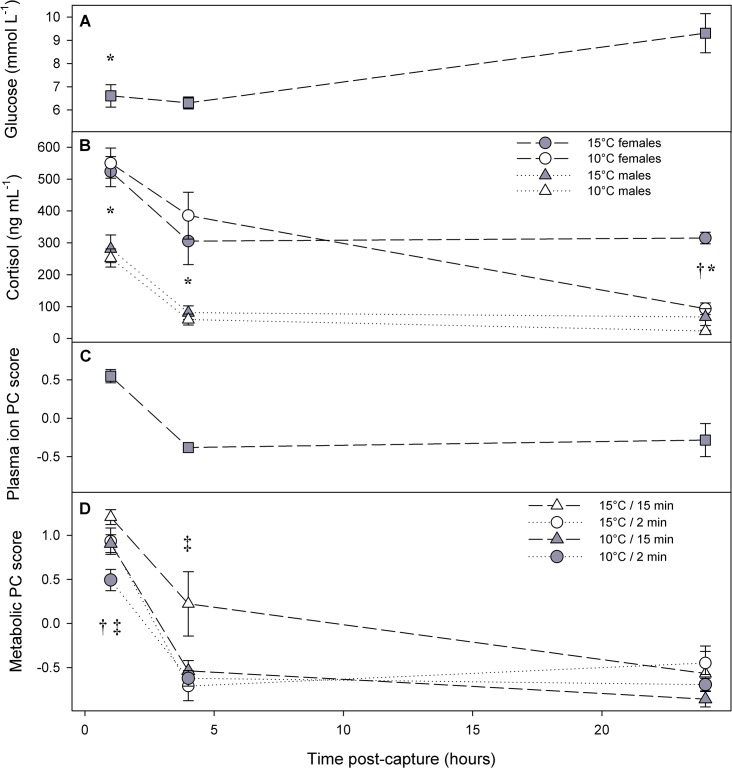
Mean ± standard error (S.E.) plasma glucose (A), cortisol (B), plasma ion PC score (C), and metabolic PC score (D) in coho salmon 1, 4, and 24 h after initiation of a 2- or 15-min seine entanglement (duration), at either 10°C or 15°C. The light grey shaded areas are mean ± standard error control values from seven fish sampled directly from the hatchery raceway (at ~7.5°C) and four fish allowed to recover from transport and handling undisturbed at CLL for 24 h (at 15°C). “Metabolic PC score” is a variable that was synthesized using principal components analysis from five original physiological variables relating to metabolic status, while plasma ion PC score was synthesized in a second PCA using three of the remaining five variables (see Table 1). For A and B, all fish were grouped because no significant differences occurred within any of the time points, except for plasma glucose at 1 h which was significantly higher in males across treatment groups (two-way ANOVA with group and sex as main effects using log-transformed data, *P* = 0.008). For B and D, significant effects within a time point are shown with * (effect of sex), † (temperature), or ‡ (entanglement duration). Separate and significant effects of temperature (*P* = 0.003) and entanglement duration (*P* = 0.004) for metabolic PC scores at 1 h were based on a two-way ANOVA (re-run after removal of a non-significant interaction term). The significant effect of entanglement duration (across both temperatures) at 4 h for metabolic PC scores (*P* = 0.035) was based on a Wilcoxon rank sum test.

In addition to sampling fish exposed to the capture simulation, seven fish were dip-netted and sacrificed directly from the hatchery raceway (~7.5°C) and a further four fish were transported to CLL and held undisturbed at 15°C for 24 h in black cylindrical fish holding bags. Data from these 11 fish were pooled to provide resting/routine values for illustrative (not statistical) purposes (light grey areas in Fig 3).

#### d. Respirometry

A subset of data logger-implanted coho salmon exposed to the capture simulation (four fish in the 10°C group and five in the 15°C group) were rapidly transferred post-simulation to 138 L static intermittent flow-through respirometers for 20–26 h of oxygen consumption rate (*Ṁ*_O2_) measurements using the same methods as Clark et al. [37]. Briefly, the respirometers recorded oxygen saturation continuously (1 Hz) using electrodes (Loligo systems, Tjele, Denmark) placed within a re-circulation line that ensured each respirometer remained well mixed. The respirometers were flushed with fresh water for 45 min every hour and sealed for the other 15 min so that *Ṁ*_O2_ could be recorded (based on the slope of oxygen saturation vs. time). Though the small sample sizes precluded statistical comparisons, respirometry allowed us to characterize post-exercise *Ṁ*_O2_ and recovery patterns for the two temperatures.

### Ethics statement

All fish transport, handling, surgical, and monitoring procedures were approved by the Animal Care committees of Fisheries and Oceans Canada and the University of British Columbia (UBC) in accordance with guidelines set by the Canadian Council on Animal Care (UBC Animal Care protocol #A11–0125). The tanks and concrete ponds in which fish were maintained were checked several times per day by members of the research team to monitor temperature, dissolved oxygen, water flow, and whether fish exhibited signs of morbidity (e.g., loss of equilibrium, erratic movement). Although this was not a survival study (i.e., we focused on physiological endpoints) eight individuals perished at some point after the end of the capture simulation but prior to set sampling times (i.e., 1, 4, and 24 h, see *Results*), which likely resulted from failure to return to homeostasis following the capture stressor, combined with the fact that natural senescence was underway for all the fish in this study. However, in this study there were no obvious signs of premature senescence or disease outbreak that can be common in experiments holding wild salmon (e.g., outbreak of Saprolegnia fungal infections; [38]). To minimize the stress that can be associated with laboratory confinement, shade cloth was suspended above each concrete pond, and sections of high-density foam were allowed to float on the water’s surface to provide cover under which most fish readily hid. Cerebral percussion was used to sacrifice all animals and was achieved by a strong, single blow to the head of the animal using a heavy wooden bat. Analgesics were not used, largely because the pharmokinetics of analgesics are poorly understood in fish and would potentially alter behaviour or physiology in ways that would interfere with the experimental objectives (i.e., assessing physiological recovery from a capture stressor).

### Laboratory analyses

To extract metabolites from white muscle samples, they were first ground to a fine powder using a mortar and pestle kept partly immersed in liquid nitrogen [25]. Approximately 0.5 g of powdered muscle was then briefly vortexed in a 15 mL falcon tube with exactly 4× the volume of perchloric acid solution (e.g., 0.5 g muscle = 2 mL solution of 8% PCA with 1 mM EDTA). The vortexed solution was incubated on ice for 10 min then centrifuged at 3000 rpm for 5 min at 4°C. The supernatant was removed and balanced to a pH of 7–8 using a neutralizing solution (2 M KOH, 0.4 M KCl, 0.3 M Imidazole), before being centrifuged at 10,000 *g* for 3 min at 4°C. The resulting supernatant was removed and stored in a -80°C freezer for later analyses of lactate, phosphocreatine (PCr), and adenosine triphosphate (ATP), which were measured in triplicate using enzymatic assays with a plate spectrophotometer (SpectraMax 340PC microplate reader with SoftMax Pro 4.8 data analysis software, Molecular Devices, Sunnyvale, CA) following details provided by Suski et al. [26].

Blood plasma was analyzed for cortisol (Neogen enzyme-linked immunosorbent assay with Spectramax 240PC plate reader, Molecular Devices, Sunnyvale, CA), chloride (Haake Buchler digital chloridometer), sodium and potassium (Cole-Palmer, model 410 single-channel flame photometer), osmolality (Advanced Instruments 3320 freezing-point osmometer), and lactate and glucose (YSI 2300 Stat Plus analyser) using methods previously detailed by Farrell et al. [25].

### Data analysis and statistics

We used principal components analysis (PCA) to integrate responses of the seven plasma and three muscle variables we measured. An initial PCA with three factors was conducted using all 10 variables (N = 155 fish) but was successively re-run after stepwise elimination of variables that either had a) a low Kaiser-Olkin-Meyer (KMO) measure of sampling adequacy (<0.5, [39]), or b) did not have a factor loading (eigenvector) ≥ |0.6| for a factor with loadings ≥ |0.6| for any other variables. That process led to a final PCA with five variables (see *Results*), from which factor scores were extracted for each fish and subsequently referred to as “metabolic PC scores”. PCA was again used on the remaining five variables, and the same process was used to refine that PCA, from which factor scores were extracted (referred to as “plasma ion PC scores”; see *Results*). Plasma cortisol and glucose, which were ultimately excluded from both PCAs (criteria described above), were analyzed separately for further analyses.

To screen for the confounding effect of sex across treatment groups for muscle and plasma variables we used a two-way analysis of variance (ANOVA) with sex and group (groups shown in Fig 2) as main effects at each time point (non-significant interaction term removed). Where sex had a significant effect, further tests for capture variables were conducted using an ANOVA with three factors (sex, entanglement duration, temperature—non-significant interactions removed). For the other variables, two-way ANOVAs were used. Data were visually examined to ensure they met parametric assumptions and tested for normality and heteroscedasticity using the Shapiro-Wilk and Levene’s test, respectively. In one case (4 h metabolic PC scores) data could not be transformed to meet parametric assumptions so Wilcoxon rank sum tests were used to separately assess overall effects of temperature and entanglement duration. Fish size (FL) was not significantly different among treatment groups within any of the three time points (two-way ANOVA with entanglement duration and temperature as factors, all *P* > 0.10).

The data logger files were downloaded into LabChart (ADInstruments, Sydney, Australia) for processing with an ECG analysis tool that was used to calculate heart rate (*f*_H_) in beats per minute. Some of the loggers failed to record data, resulting in a final N of 21–5 each for 10°C/2 min and 10°C/15 min, 2 for 15°C/2 min, and 9 for the 15°C/15 min treatment. All ECG data were manually examined to ensure correct beat counting. For each fish, we assessed its baseline (resting) *f*_H_ by averaging over a ~15 h period preceding the capture simulation. In some cases, certain sections were excluded or periods greater than 15 h prior to capture were used to ensure baseline for each fish was calculated from a minimum of 10 h of low, stable *f*_H_ data. Time to recovery after the fisheries simulation was assessed as the time point when *f*_H_ returned to within the 99% confidence interval (CI) of that individual’s baseline *f*_H_ (mean ± standard deviation CI = 1.39 ± 0.80 beats min^-1^). We also calculated *f*_H_ elevation for each fish by subtracting its baseline *f*_H_ + 99% CI from its raw *f*_H_ at each time point, such that when *f*_H_ elevation decreased to ≤ 0 a fish was considered “recovered”. Heart rate elevation was used to calculate excess post-stressor heart beats (EPHB) for each fish by integrating the area under the recovery curve (until recovery was reached or until the fish was sacrificed if it did not recover within 24 h) using the simple trapezoidal method.

Effects of entanglement duration and temperature on post-capture peak *f*_H,_ *f*_H_ elevation, the factorial increase in *f*_H_, recovery time, and EPHB were assessed using two-way ANOVAs using type III sums of squares with the interaction term removed because of unbalanced sample sizes. To assess whether capture variables resulted in relative elevations in *f*_H_ during an extended recovery, we used a linear mixed effects model with entanglement duration, temperature, and time as fixed effects, *f*_H_ elevation as the outcome variable, and fish ID as a random variable, focusing on *f*_H_ at 10, 15, and 20 h post-capture. Those time points were chosen to attempt to understand causes of extended *f*_H_ elevation, and because it was apparent that there was little among-group variation in the initial response and recovery (e.g., up to 5–7 h). Statistics were conducted using RStudio (v. 0.98.953, RStudio, Inc., Boston, MA, USA; http://www.rstudio.com/). Tests were assessed as significant at α = 0.05 and data are presented as mean ± standard error.

## Results

### Blood and white muscle physiology

The only fish to die in the experiment came from the most stressful group, 15-min entanglement treatment at 15°C, where 18% of fish died after release but prior to sampling (four in the 1 h group, three in the 4 h group, and one in the 24 h group; samples not included in analyses).

Fish exhibited an elevation in indices of exhaustion and stress 1 h after initiation of the stressor (Fig 3). Lactate was elevated in plasma and white muscle relative to reference and 24 h values, as was plasma cortisol, while ATP and PCr were depressed (Fig 3; data for individual variables available in online appendix). Metabolic indices were well-integrated by PCA (Table 1); the synthetic “metabolic PC scores” variable positively correlated with plasma and muscle lactate, and osmolality, and negatively correlated with muscle ATP and PCr. Overall, metabolic PC scores were elevated at 1 h but decreased to resting/routine (light grey areas in Fig 3) values by 4 h for most fish (Fig 3D). At 1 h, there were significant and separate positive effects of entanglement duration (Two-way ANOVA; *F*_1,44_ = 9.5, *P* = 0.004) and water temperature (*F*_1,44_ = 10.3, *P* = 0.003) on metabolic PC scores (Fig 3D). At 4 h, metabolic PC scores were significantly elevated across temperatures for fish exposed to the longer entanglement duration (Wilcoxon rank sum test, *P* = 0.034), whereas there was no apparent effect of temperature itself (*P* = 0.29). A Kruskal-Wallis ANOVA failed to detect a significant difference among the four groups shown in Fig 2D (χ^2^ = 7.8, df = 3, *P* = 0.051). By 24 h, there were no significant differences among groups in metabolic PC scores, with all groups apparently recovered (Two-way ANOVA, *P* > 0.40 for both entanglement duration and temperature).

**Table 1:** Output of two separate principal components analyses (PCA; N = 155 individuals) whose resulting factor scores (PC1) were used for statistical analyses (see Fig 3).

First PCA—metabolic PC score		
Tissue variable	**PC1** loading	Communality (h^2^)
Eigenvalue	3.86	
Variance explained	77%	
Plasma lactate	**0.91**	0.83
Plasma osmolality	**0.89**	0.79
Muscle lactate	**0.94**	0.88
Muscle ATP	**-0.85**	0.73
Muscle PCr	**-0.80**	0.63
Second PCA—plasma ion PC score		
	**PC1** loading	Communality (h^2^)
Eigenvalue	2.23	
Variance explained	74%	
Plasma Cl^-^	**0.91**	0.83
Plasma K^+^	**-0.78**	0.61
Plasma Na^+^	**0.89**	0.79

The first PCA, whose resulting factor scores are referred to as metabolic PC (principal component) scores, resulted after an initial PCA with all ten original physiological metrics, from which variables were successively removed because of having either a) a low Kaiser-Mayer-Olkin (KMO) measure of sampling adequacy (see Field et al. 2012), or b) not having a loading ≥ |0.6| (shown in bold) for any factor which also had other ≥ |0.6| loadings (i.ef., not agreeing strongly with other variables within a factor). PC loadings represent correlation coefficients (*r*) between the original variable and the new synthetic (e.g., PC1) variable. The second PCA (bottom) whose resulting factor scores are referred to as plasma ion PC scores, was initially run using the five remaining variables, and was simplified after the same iterative procedure used to refine the first PCA.

A second PCA on the remaining five variables strongly integrated the three plasma ions (chloride, potassium, and sodium) into a synthetic variable referred to as “plasma ion PC scores” (Table 1), which similarly showed signs of elevation at 1 h followed by a decrease at 4 h (Fig 3C). There were no significant effects of treatment variables on recovering plasma ion PC scores (thus, all fish grouped in Fig 3C). Likewise, plasma glucose was not significantly affected by capture variables at the three time points (all *P* > 0.40), but was significantly higher in males across groups at 1 h after capture (Two-way ANOVA, sex *F*_1,43_ = 7.7, *P* = 0.008). Cortisol (log_10_-transformed) was higher in females across time points (*P* < 0.001). Controlling for sex, plasma cortisol was not affected by temperature or entanglement duration at 1 or 4 h, but after 24 h there was a significant positive effect of temperature (*F*_1,40_ = 8.74, *P* = 0.004), in addition to a separate effect of sex (*F*_1,40_ = 13.43, *P* = 0.002; using a three-way ANOVA, interactions removed due to non-significance; Fig 3B).

### Cardiorespiratory recovery

**Fig 4:**
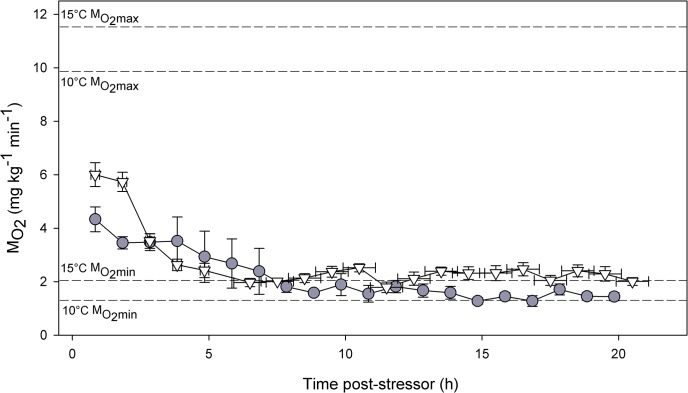
Mean ± S.E. oxygen consumption rate (*Ṁ*_O2_) for fish recovered in static respirometers after the capture simulation at 10°C (grey circles) and 15°C (white triangles). The total sample size was five individuals at 15°C (two following 2 min of entanglement, three following 15 min) and four at 10°C (all 15 min entanglement time) but the sample size was smaller for some time points where individuals exhibited spontaneous activity, reflected by sudden increases in *Ṁ*_**O2**_ (and confirmed by visual observation of respirometry chambers). Temperature-specific maximum (*Ṁ*_**O2max**_) and minimum (*Ṁ*_**O2min**_) aerobic metabolic rates for this population of coho salmon were measured in a separate experiment and are shown using dashed lines for illustrative purposes—maxima were obtained using a Brett-type swim tunnel respirometer (G.D. Raby, *unpublished data*).

Confinement in a respirometer for 24 h of hourly *Ṁ*_O2_ measurements elicited periodic bouts of visually observable activity that were reflected in spikes in *Ṁ*_O2_ among the small number of fish placed in respirometers (N = 5 for 15°C, N = 4 for 10°C). Those data points were removed for a characterization of the respiratory recovery following our capture simulation (Fig 4). In the first post-release measurements (mean 0.84 h after initiation of the stressor, ~ 0.54 h after release), *Ṁ*_O2_ reached 4.33 ± 0.45 mg kg^-1^ min^-1^ for fish in the 10°C treatment and 6.00 ± 0.45 mg kg^-1^ min^-1^ at 15°C; ~50% of *Ṁ*_O2max_ for both temperatures in this population of coho salmon (G.D. Raby, *unpublished data*; Fig 4). *Ṁ*_O2_ returned to resting values quicker in the 15°C treatment (~5 h) than at 10°C (~8 h). Small sample sizes within time points precluded statistical analyses of *Ṁ*_O2_ data.

**Fig 5:**
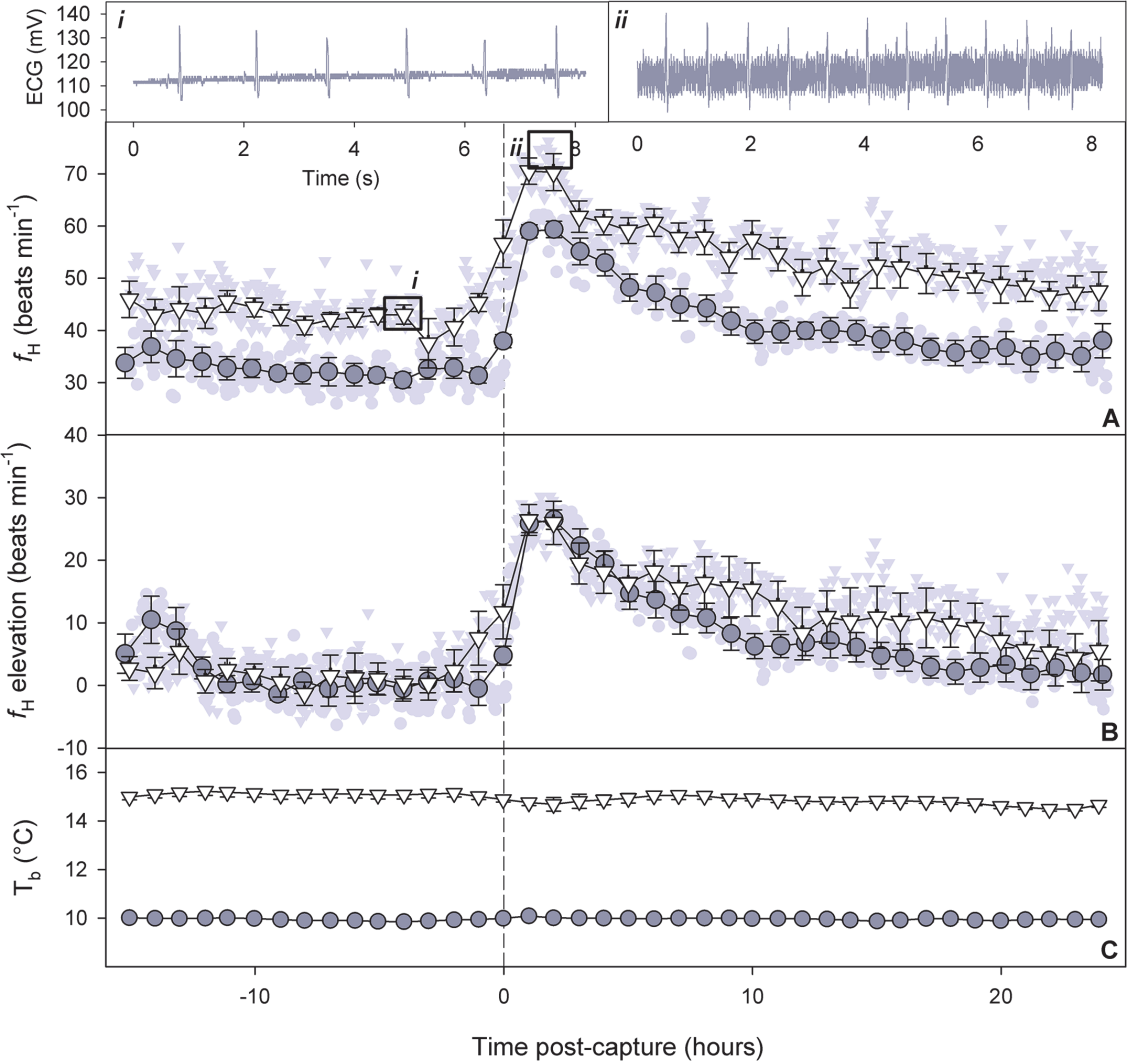
Mean heart rate (*f*_H_) (A), heart rate elevation relative to baseline (B), and body temperature (T_b_) (C) for coho salmon implanted with data loggers and subject to fisheries simulations at 10°C (grey circles) and 15°C (white triangles). Hourly means are based on data within ~30 min of each hour mark and error bars represent standard error (x-axis error bars present but too small to be visible in most cases; likewise for y-axis error for T_**b**_ in the 10°C group). Background data points in light grey are group means for each time point (i.e., every 6 min; triangles for 15°C, circles for 10°C). Heart rate relative to baseline (*f*_**H**_ elevation; B) was calculated for each individual by subtracting that individual’s baseline from its actual heart rate at a given time point. Also shown in the top panels are sample electrocardiogram (ECG) traces (recorded at 200 Hz) used to assess heart rate from a logger-implanted salmon held at 15°C (*i* = baseline, *ii* = post-capture).

Heart rate (*f*_H_) baseline and post-capture peak values reflected temperature differences but were not affected by entanglement duration (Fig 5). Baseline *f*_H_ was 32.3 ± 1.5 beats min^-1^ at 10°C, which was significantly lower than at 15°C where it averaged 42.2 ± 1.4 beats min^-1^ (Welch’s t-test, t_17.95_ = -4.93, P < 0.001). After capture, *f*_H_ peaked at 67.9 ± 1.3 (10°C) and 85.5 ± 1.7 beats min^-1^ (15°C) and the difference between temperatures was significant (Table 2). Net *f*_H_ elevation (Fig 5B) was also significantly higher at 15°C than at 10°C, but there was no difference in the factorial increase, with heart rate approximately doubling at both temperatures (Table 2). Post-capture peak *f*_H_, peak *f*_H_ elevation above baseline, and factorial elevation were not significantly affected by entanglement duration (Fig 5 and Table 2). Likewise, after controlling for capture variables, sex had no effect on any of the above heart rate metrics (all *P* > 0.08).

**Table 2:** Comparative summary of heart rate (*f*_H_) responses and recovery among the four capture treatments.

Temperature, entanglement duration	N	Post-capture peak *f*_H_ (beats min-1)	Peak *f*_H_ elevation (relative to baseline)	Factorial post-capture *f*_H_ increase	Recovered to baseline *f*_H_ within 24 h	Recovery time (h) for fish that recovered	Excess post-capture heart beats
10°C							
2 min	5	67.9 ± 1.8	35.6 ± 2.3	2.2 ± 0.1	40% (2 of 5)	13.7 ± 0.3	18628 ± 2189
15 min	5	67.9 ± 2.1	33.1 ± 0.8	2.1 ± 0.1	100% (5 of 5)	8.5 ± 1.3	7421 ± 1348
15°C							
2 min	2	86.6 ± 3.3	39.1 ± 4.5	2.0 ± 0.2	100% (2 of 2)	5.9 ± 2.0	3785 ± 1418
15 min	9	85.3 ± 1.9	41.9 ± 2.3	2.1 ± 0.1	56% (5 of 9)	17.6 ± 2.2	23439 ± 4222
Statistics^1^							
Temperature		*F*_1,18_ = 62.75, *P* < 0.001	*F*_1,18_ = 6.92, *P* = 0.02	*F*_1,18_ = 0.50, *P* = 0.49	*Z* = –1.19, *P* = 0.23	*F*_1,11_ = 0.70, P = 0.42	*F*_1,18_ = 1.50, P = 0.24
Duration		*F*_1,18_ = 0.05, *P* = 0.84	*F*_1,18_ = 0.02, *P* = 0.89	*F*_1,18_ = 0.03, *P* = 0.86	*Z* = –1.20, *P* = 0.23	*F*_1,11_ = 1.01, *P* = 0.34	*F*_1,18_ = 0.03, *P* = 0.86

Values represent mean ± standard error. Notes: *f*_H_ elevation relative to baseline, factorial increase, and *f*_H_ recovery were assessed for each individual relative to that individual’s unique baseline *f*_H_. Factorial increase in *f*_H_ was calculated by dividing an individual’s peak post-capture heart rate by its pre-capture baseline average. Total excess post-capture heart beats (EPHB) was estimated by integrating the total area under the recovery curve for each fish (to recovery or 24 h in the case of fish that did not return to baseline before being sacrificed at 24 h).

^1^Two-factor ANOVAs (no interaction term) used except for numbers of fish recovering to baseline within 24 h, for which a multiple logistic regression was used.

Heart rate recovered to baseline levels notably slower than *Ṁ*_O2_ and other metrics measured in this study (Fig 5B). Overall, 7 of 21 fish did not return to their pre-capture baseline *f*_H_ within the 24 h after release. Among the 14 fish that did recover before being sacrificed, recovery time ranged from 3.1 to 22.5 h. Effects of entanglement duration and temperature on EPHB, likelihood of recovery within 24 h, and recovery time were not significant (Table 2). A linear mixed-effects model examining heart rate elevation (Fig 4B) across three time points (10, 15, and 20 h) with fish ID as a random variable revealed a significant negative effect of time (i.e., heart rate declined across the time points examined—evident in Fig 5B; *β* = -0.48 ± 0.14, *t*_39_ = -3.44, *P* = 0.001), but no significant effects of temperature (*t*_17_ = 0.52, *P* = 0.61) or entanglement duration (*t*_17_ = 0.98, *P* = 0.34).

## Discussion

### Physiological responses

Both warmer water and longer net entanglement result in greater physiological disturbance in coho salmon and, for some variables, an extended recovery after simulated fisheries capture based on the data presented here. White muscle and plasma variables provided the strongest evidence of treatment effects (Fig 3). Overall, we found that most physiological variables had recovered or approached routine levels within 4 h (i.e., 4 h, Figs 3 and 4) but that many individuals took much longer to return to pre-stressor *f*_H_ (Fig 5 and Table 2).

In a closely related population of coho salmon held at ~8°C, Donaldson et al. [28] found that *f*_H_ took up to 16 h to recover from an exhaustive exercise stressor, while Anderson et al. [30] similarly found Atlantic salmon required 15 h to recover *f*_H_ after angling (at 8 and 16.5°C). In contrast are centrarchids (Centrarchidae), which return to routine *f*_H_ within 2–4 h of exercise and angling stressors [17,40,41]. Our data support previous findings that the relative increase in *f*_H_ from fisheries-related stressors is not affected by the nature of the stressor or water temperature, with an approximate doubling of *f*_H_ in all cases [28,40]. However, whereas the duration of air exposure has a strong effect on *f*_H_ recovery time in rock bass (*Ambloplites rupestris*; [40]), we found the duration of net entanglement had no such effect in salmon, though our ability to detect such effects was somewhat limited by low statistical power. More notable in our study was that several fish did not return to resting *f*_H_ within 24 h, and that recovery time varied widely even within treatments. As such, our data confirm that capture-related stressors can cause very prolonged *f*_H_ elevation in free-swimming salmon. Accordingly, we suggest that *f*_H_ may provide the best indication of whole-organism recovery from a stressor but not of the severity of the stressor. Future research could explore why salmon exposed to seemingly identical acute stressors can vary so widely in their recovery profiles—knowledge that could help explain why delayed mortality occurs. Such differences could potentially be explained by inter-individual variation in spontaneous activity levels, stress responsiveness (e.g., [42,43]), physiological or behavioural syndromes [44], prior experiences (e.g., “training”, [45], or “carry-over effects”, [46]), or pre-existing pathogen loads [8].

The biological importance of an extended elevation in *f*_H_ (e.g., 15+ h) following capture remains unclear, particularly in light of the fact that *Ṁ*_O2_ appeared to return to baseline relatively quickly (Fig 4), meaning the direct energetic cost of recovery was modest. Based on the mean recovery profiles in *Ṁ*_O2_ in Fig 4, excess post-exercise oxygen consumption was 783 ± 284 and 501 ± 98 mg O_2_ kg^-1^ at 10 and 15°C, respectively. Those values translate to 2358 ± 920 and 1623 ± 317 calories of excess energy used during recovery from capture; energy that could otherwise be used to achieve 1.4 ± 0.2 km (15°C) to 2.2 ± 0.8 km (10°C) of upstream migration, or 1.1 ± 0.2 h (15°C) to 1.8 ± 0.6 h (10°C) of spawning activity (based on migration energetics data in [47]). Perhaps a more important energetic consideration was the mismatch between oxygen demand and availability during entanglement. Both in the actual fishery and our simulation, dissolved oxygen declined from 9–10 mg L^-1^ (~90–100%) to ~ 5–7 mg L^-1^ (50–70%) in the crowded seine within 10–15 min, while oxygen demand (Fig 4) ranged from ~ 8–24 mg min^-1^ per fish (depending on body size and water temperature), likely necessitating a significant shift towards anaerobic metabolism.

Reliance on anaerobic metabolism can explain why the fisheries capture simulation caused changes in blood and muscle metrics that were modulated by temperature and the duration of net entanglement. The initial corralling and entanglement elicited ~ 1 min of exercise, which was followed by 2–15 min of crowding in very shallow water with declining oxygen content (e.g., 60% air saturation within 10 min). The protocol was more typical of a true fisheries net capture than those applied by exhaustive exercise studies (e.g., 5 min of manual chasing; [16]). Nevertheless, the rich physiological literature that exists on the latter [23] is relevant to understanding our results. Anaerobic exercise relies initially on using white muscle stores of PCr and ATP (whose concentrations remain unchanged during aerobic swimming; [48,49]), and thereafter shifts to greater consumption of glycogen (glycogenolysis), resulting in production of lactate and a drop in pH [48]. Some lactate leaks out of muscle cells to blood plasma [49], while decreased muscle pH creates an osmotic pull of water from plasma to muscle cells, effectively concentrating plasma ions (i.e., heightened osmolality and plasma ion PC scores at 1 h; S1 Table and Fig 3). ATP and PCr in muscle typically recover to resting levels within 1–2 h of exhaustion [26,49], while muscle lactate conversion back to glycogen (glycogenesis; [49]), the primary fate of muscle lactate, typically occurs more slowly (e.g., 6–8 h), as does the restoration of osmotic balance [50]. These processes were effectively integrated into a synthetic variable (metabolic PC score) for our experiment, which can be thought of as a robust measure of departure from metabolic homeostasis, where higher scores represent more exhausted fish and low scores represent a rested state (Table 1 and Fig 3). Although we did not measure muscle glycogen, we expect that it would have inversely tracked lactate [49]. It was notable that although fish likely did not exercise maximally, evidenced by only reaching 50% of maximum attainable *Ṁ*_O2_, lactate reached maximal levels comparable to Atlantic salmon and rainbow trout in exhaustive exercise experiments (i.e., ~ 40 and ~20 mmol L^-1^ in muscle and blood plasma, respectively; [15,16]), but only in the 15 min duration entanglement (Fig 3; S1 Table). Entanglement time had a significant effect on metabolic disturbance, which helps explain why it was negatively correlated with reflex impairment in the field [32], and is supported by the catch-and-release literature where longer angling or air exposure have both been shown to cause greater lactate accumulation and longer recovery of cardiac variables [17,40,51,52].

Temperature had a significant effect on metabolic PC scores at 1 h, likely resulting from a combination of higher metabolic rate at 15°C (Fig 4) and to a lesser extent the lower dissolved oxygen content of warmer water. Although past studies on the effects of temperature on physiological responses to exhaustive exercise have found minimal differences in resulting lactate loads [15,16], the present study exposed fish to hypoxia which only followed brief burst swimming during netting. Therefore, exhaustion was likely to be a function of both entanglement time and temperature, given the role of the latter in determining metabolic rate (Fig 4). In fact, it appears fish were not fully exhausted in the 2-min duration treatments based on their lower muscle lactate loads (and metabolic PC scores) at 1 h (Fig 3). After exhaustive exercise, largemouth bass (*Micropterus salmoides*) accumulate more lactate and have more depressed white muscle energy stores if recovered in hypoxic or warmer water [26]. Similar trends have been observed in bonefish (*Albula vulpes*, [53]). Importantly, resting levels of ATP, PCr, and glycogen are relatively independent of acclimation temperature in fish ([16]), such that exposure to hypoxia should deplete energy stores more quickly at a higher temperature (Fig 4, [26]). In addition, the crowded fish would have depleted the oxygen content of the water more quickly, which is consistent with mortality occurring only in the 15°C/15 min treatment. The fish that survived the 15°C/15 min treatment exhibited the highest mean metabolic PC scores at 1 and 4 h, the longest heart rate recovery, and the highest number of excess post-stressor heartbeats (Table 2), although statistically significant differences did not occur in the latter two cases. Collectively, our data show that physiological disturbance in coho salmon is increased by longer entanglement time, particularly in warmer water.

A prediction from the literature is that physiological recovery from exhaustion in fish is more rapid in warmer water [15]. The data from our experiment generally did not support that prediction, with the exception of our small *Ṁ*_O2_ dataset. However, the focus of our sampling design on a small number of time points, in the case of blood and muscle variables, likely precluded our ability to detect such effects. Interestingly, there was an effect of temperature on plasma cortisol recovery, with cortisol remaining particularly high in females in the 15°C treatment 24 h after release (~ 300 ng mL^-1^; Fig 3). Though we have little pre-capture control data (plasma cortisol was 15.9 and 131.2 ng mL^-1^ in two 15°C female controls [among four 15°C controls]; S1 Table), the data suggest either a) female salmon may have impaired recovery of cortisol after capture in warm water, or b) cortisol is maintained at higher routine levels in warmer water, though this is not the case in sockeye or pink salmon [38]. It has recently been established that mortality is exceptionally high in upriver migrating female sockeye salmon exposed to warm water and capture stressors [6,38,54,55], and our cortisol data here may help explain why those trends occur. Integrating knowledge about sex-specific consequences of fisheries capture is particularly relevant to salmon populations in light of warming river temperatures because females are usually the limiting sex to spawning ground productivity [56]. Further experiments are required to establish whether impaired cortisol recovery is a mechanism for delayed sex-specific mortality of salmon caught and released in warm water.

### Relevance to conservation and management

Our data are directly relevant to understanding bycatch of endangered coho salmon in Fraser River beach seine fisheries. Beach seine fisheries that encounter coho salmon typically occur in mid-September (targeting pink and sockeye salmon) when water temperatures are 14–16°C [32], but the fishery also sometimes re-opens in late October when water temperatures are 8–10°C (to target chum salmon). It has been proposed that upriver migrating salmon are adapted to the modal water temperatures they historically experience at the time of river entry [57] and in our case we used a population of salmon for which 15°C would represent the upper limit of their lifetime experience, with 10°C being closer to their modal upriver migration temperature (water was 8–10°C in the Chilliwack River at the time of the experiment). In the fishery, where entanglement times ranged from 5 s to 56 min (median = 3.3 min; [32]) plasma lactate averaged 12.3mmol L^-1^, ~8–10 min after release from the net [35]. This is only slightly lower than our 1 h samples, which were taken closer to when plasma lactate peaks during recovery [25,58]. Thus, although in an experimental setting and using a surrogate population, our data are relevant to informing best handling practices for the beach seine fishery, and help highlight the importance of releasing bycatch from nets as rapidly as possible [32], particularly at elevated temperatures.

In a human dimensions survey of fishery participants, rapid release of coho salmon was the most commonly suggested method for reducing bycatch mortality. Many fishers (35%), however, presented no ideas for reducing mortality, suggesting there is potential to increase awareness of the importance of rapid release [32]. Our experiment illustrates that the mismatch between oxygen demand and supply during crowding results in additional and substantial physiological disturbances (Fig 3); clear evidence of why, if rapid release is not possible, fishers should be urged to maximize oxygen availability by leaving the net in deeper water for sorting. Most fishers that participated in the survey [32] indicated a willingness to leave their nets in knee-deep water, although the depth required to ensure crowding does not deplete dissolved oxygen would likely depend on a variety of factors, such as catch size and local water flow.

In addition to informing bycatch management for an endangered population of salmon, the trends relating to handling time and temperature in our experiment are likely applicable to other fisheries. For example, similar physiological data and recommendations exist in rainbow trout recreational fisheries [21] and fyke net fisheries that bycatch northern pike [59]. Our maximal and recovered muscle lactate data are comparable to those that occur in coho salmon captured in marine gillnet and troll fisheries [25,34], further emphasizing that our data are likely replicable and relevant in real fisheries.

Estimates of global marine fisheries bycatch range from 6.8 to 38.5 million tonnes [60,61]—a conservation problem that has caused population declines (e.g., [62,63]) and drawn considerable research effort in the last 20 years [64]. In Canada’s Pacific fisheries, a policy to move towards “selective fishing” has been in place for more than ten years, which states that non-target fish should be released “unharmed” if bycatch cannot be avoided [9]. Our experiment is relevant in this context, given that physiological data are objective measures of fish welfare [65]. To date, there is sparse use of terms relating to stress, welfare, or the sublethal effects of bycatch in IUCN documents on imperiled species that are captured in commercial fisheries [31]. Nevertheless, well-controlled experiments with physiological assessments can help provide mechanisms needed to facilitate evidence-based implementation of best practices [66], especially when complemented by field and human dimensions data (e.g., [67]). We hope the present study provides a helpful addition to a growing physiological literature (for reviews see [24,31,68]) that can be used by conservation practitioners to understand trends in fish impairment and mortality while moving towards methods of live release that benefit the welfare and survival of bycatch.

## Supporting Information

S1 Table Mean ± standard error (range) blood plasma and white muscle measures for each treatment group (water temperature, capture stressor duration) at different durations after initiation of the capture stressor. Results obtained from mortalities are for illustrative purposes only—those fish perished at an unknown time between the capture stressor at that sampling time (i.e., 1 h or 4 h), and some of the measured constituents may have rapidly broken down following death. 24 h controls are fish that were transported to CLL and placed in black flow-through fish bags for 24 h before being rapidly sacrificed and sampled for tissue. 24 h control values were combined with those from the hatchery raceway to provide the control levels in Fig 3 (grey shaded areas).
(DOCX)
