# Supplementary material for: Facing the River Gauntlet: Understanding the Effects of Fisheries Capture and Water Temperature on the Physiology of Coho Salmon
Source: PLoS One. 2015 Apr 22;10(4):e0124023. doi: 10.1371/journal.pone.0124023 (PMC4406555; doi:10.1371/journal.pone.0124023)
Supplement: S1 Table — Results obtained from mortalities are for illustrative purposes only—those fish perished at an unknown time between the capture stressor at that sampling time (i.e., 1 h or 4 h), and some of the measured constituents may have rapidly broken down following death. 24 h controls are fish that were transported to CLL and placed in black flow-through fish bags for 24 h before being rapidly sacrificed and sampled for tissue. 24 h control values were combined with those from the hatchery raceway to provide the control levels in Fig 3 (grey shaded areas). (DOCX) [file pone.0124023.s001.docx]

**Supporting Information**

**Table S1. Mean ± standard error (range) blood plasma and white muscle measures for each treatment group (water temperature, capture stressor duration) at different durations after initiation of the capture stressor.**

|  |  | |  | BLOOD PLASMA | | | | | | | WHITE MUSCLE | | |
| --- | --- | --- | --- | --- | --- | --- | --- | --- | --- | --- | --- | --- | --- |
| Post-capture time | Treatment | | n | Glucose (mmol L^-1^) | Cortisol (ng mL^-1^) | Na^+^  (mmol L^-1^) | K^+^  (mmol L^-1^) | Cl^-^  (mmol L^-1^) | Osmolality (mOsm kg^-1^) | Lactate (mmol L^-1^) | Lactate (mmol L^-1^) | PCr  (µmol  g^-1^) | ATP (µmol g^-1^) |
| 1 h | 10°C | 15 min | 14 | 6.3 ± 0.5  (4.0–9.0) | 483 ± 64  (181–902) | 153.0 ± 1.5 (143–163) | 3.14 ± 0.25 (1.81–5.10) | 132.4 ± 1.0 (125–138) | 349.6 ± 2.9  (332–369) | 15.4 ± 0.9 (10.5-20.0) | 38.5 ± 1.5 (28.0-48.0) | 11.2 ± 1.5  (4.1-22.2) | 3.7 ± 0.4  (1.7-7.0) |
|  |  | 2 min | 12 | 7.0 ± 0.7  (4.8–14.8) | 430 ± 58  (135–759) | 148.2 ± 2.7  (129–169) | 3.28 ± 0.32  (1.41–4.98) | 128.7 ± 2.0  (111–135) | 334.1 ± 4.0  (316–357) | 12.2 ± 0.8  (7.6–17.1) | 33.2 ± 1.5 (23.8–40.0) | 7.4 ± 1.2 (1.2–16.1) | 5.1 ± 0.4 (3.0–7.8) |
|  | 15°C | 15 min | 12 | 6.8 ± 1.0 (3.4–14.4) | 469 ± 79 (129–898) | 154.3 ± 1.3 (145–162) | 3.68 ± 0.56  (1.66–8.72) | 126.9 ± 2.0 (113–139) | 357.1 ± 3.0 (331–371) | 20.8 ± 1.2 (13.3–28.2) | 39.5 ± 1.1 (33.1–45.0) | 9.4 ± 1.8 (0.9–16.7) | 3.8 ± 0.4 (1.6–5.5) |
|  |  | 2 min | 10 | 6.0 ± 0.5 (4.0–8.3) | 347 ± 44 (166–616) | 151.7 ± 2.8 (137–167) | 4.38 ± 0.40 (1.71–6.64) | 131.0 ± 1.3 (124–138) | 345.6 ± 4.2 (315–361) | 16.8 ± 1.3 (7.3 – 21.0) | 37.2 ± 1.5 (25.1–42.5) | 6.6 ± 1.1 (1.6–11.5) | 4.6 ± 0.4 (3.3–6.7) |
| 4 h | 10°C | 15 min | 13 | 6.4 ± 0.5 (4.4–9.9) | 329 ± 93 (21–1138) | 137.7 ± 0.9 (133–143) | 5.32 ± 0.57 (1.51–9.30) | 116.5 ± 1.5 (103–124) | 307.6 ± 1.9 (299–327) | 7.5 ± 1.4 (1.9–18.7) | 20.6 ± 1.3 (12.7–27.8) | 16.1 ± 1.6 (3.9–25.2) | 6.3 ± 0.7 (3.9–13.5) |
|  |  | 2 min | 12 | 6.3 ± 0.5 (3.8–9.8) | 202 ± 61 (16–703) | 137.6 ± 1.7 (130–148) | 4.71 ± 0.46 (2.31–7.80) | 120.7 ± 1.9 (108–131) | 308.3 ± 2.3 (297–319) | 3.5 ± 0.6 (0.8–7.3) | 23.0 ± 1.2 (15.9–30.3) | 15.7 ± 1.0 (8.1–19.7) | 6.8 ± 0.3 (4.6–8.4) |
|  | 15°C | 15 min | 10 | 5.8 ± 0.8 (1.3–9.1) | 216 ± 61 (13–607) | 143.3 ± 1.5 (136–152) | 6.77 ± 0.70 (4.57–11.01) | 118.5 ± 1.3 (113–125) | 318.2 ± 4.6 (299–344) | 14.2 ± 2.1 (6.6–26.3) | 28.9 ± 4.7 (15.8–57.0) | 13.0 ± 2.5 (0.7–23.5) | 5.2 ± 0.9 (0.2–7.5) |
|  |  | 2 min | 11 | 6.6 ± 0.4 (5.0–9.4) | 212 ± 46 (47–517) | 140.4 ± 1.7 (134–152) | 5.21 ± 0.41 (3.52–7.57) | 119.2 ± 1.3 (111–127) | 310.8 ± 2.2 (297–321) | 5.9 ± 1.1 (2.0–14.2) | 20.3 ± 1.2 (15.7–27.3) | 17.8 ± 1.8 (9.3–31.1) | 7.3 ± 0.8 (4.4–14.6 |
| 24 h | 10°C | 15 min | 4 | 7.7 ± 1.0 (5.8–9.5) | 114 ± 42 (27–221) | 139.1 ± 3.4 (130–146) | 4.00 ± 0.62 (2.41 – 5.08) | 123.0 ± 2.3 (120–130) | 305.4 ± 2.5 (301–312) | 1.7 ± 0.3 (1.2–2.4) | 20.8 ± 2.3 (14.2–25.2) | 15.5 ± 1.8 (13.0–20.6) | 6.5 ± 0.2 (6.1–6.9) |

(Table S1 continues.)

|  |  |  |  | BLOOD PLASMA | | | | | | | WHITE MUSCLE | | |
| --- | --- | --- | --- | --- | --- | --- | --- | --- | --- | --- | --- | --- | --- |
| Post-capture time | Treatment | | n | Glucose (mmol L^-1^) | Cortisol (ng mL^-1^) | Na^+^  (mmol L^-1^) | K^+^  (mmol L^-1^) | Cl^-^  (mmol L^-1^) | Osmolality (mOsm kg^-1^) | Lactate (mmol L^-1^) | Lactate (mmol L^-1^) | PCr  (µmol  g^-1^) | ATP (µmol g^-1^) |
| 24 h |  | 2 min | 10 | 10.1 ± 1.3 (6.0–19.2) | 79 ± 24 (13–216) | 137.5 ± 1.6 (128–145) | 3.80 ± 0.30 (2.56–5.30) | 122.2 ± 2.3 (112–132) | 306.0 ± 1.4 (300–316) | 1.9 ± 0.5 (0.6–5.8) | 20.7 ± 1.5 (15.4–28.3) | 15.7 ± 0.8 (11.4–19.8) | 6.2 ± 0.4 (4.2–8.0) |
|  | 15°C | 15 min | 14 | 11.2 ± 2.4 (0.4 – 32) | 312 ± 88 (17–1126) | 131.2 ± 4.6 (75–146) | 8.90 ± 4.36 (2.60–65.37) | 115.9 ± 4.7 (61–130) | 311.9 ± 7.8 (293–412) | 4.7 ± 2.7 (1.1–39.5) | 21.1 ± 2.3 (14.8–46.3) | 17.1 ± 1.7 (0.1–25.3) | 6.3 ± 0.5 (0.1–7.8) |
|  |  | 2 min | 13 | 7.5 ± 1.0 (4.3–16.9) | 133 ± 41 (13–464) | 141.6 ± 1.8 (126–152) | 4.36 ± 0.43 (1.91–7.47) | 129.5 ± 1.5 (121–136) | 314.1 ± 1.3 (305–321) | 2.1 ± 0.3 (0.5–4.5) | 23.8 ± 1.4 (15.9–31.4) | 13.7 ± 1.7 (1.3–21.9) | 6.7 ± 0.4 (4.6–8.3) |
| 24 h controls | 15°C | | 4 | 6.0 ± 0.6 (4.3–6.8) | 47 ± 28 (16–131) | 144.1 ± 1.5 (142–149) | 4.39 ± 0.85 (2.73–6.76) | 128.9 ± 1.2 (126–132) | 310.1 ± 2.2 (306–314) | 1.6 ± 0.5 (0.7–2.6) | 25.1 ± 3.4 (19.1–30.9) | 12.4 ± 3.1 (7.4–18.1) | 5.7 ± 0.6 (4.9–6.8) |
| Hatchery raceway controls | 7.5°C | | 7 | 7.3 ± 1.5 (3.9–14.3) | 144 ± 60 (13–4.6) | 140.8 ± 3.0 (124–150) | 3.07 ± 0.22 (2.42–4.00) | 124.6 ± 4.9 (95.4–134.9) | 305.2 ± 4.9 (273–316) | 1.6 ± 0.7 (0.3–6.1) | 18.7 ± 2.0 (13.5–27.5) | 16.7 ± 1.5 (12.0–23.8) | 6.3 ± 0.5 (4.7–7.6) |
